# Exploring Levansucrase Operon Regulating Levan-Type Fructooligosaccharides (L-FOSs) Production in *Priestia koreensis* HL12

**DOI:** 10.4014/jmb.2404.04043

**Published:** 2024-08-23

**Authors:** Hataikarn Lekakarn, Daran Prongjit, Wuttichai Mhuantong, Srisakul Trakarnpaiboon, Benjarat Bunterngsook

**Affiliations:** 1 Department of Biotechnology, Faculty of Science and Technology, Thammasat University, Rangsit Campus, Khlong Nueang, Khlong Luang, Pathum Thani 12120, Thailand; 2 Enzyme Technology Research Team, Biorefinery Technology and Bioproduct Research Group, National Center for Genetic Engineering and Biotechnology, 113 Thailand Science Park, Phahonyothin Road, Khlong Nueang, Khlong Luang, Pathum Thani 12120, Thailand

**Keywords:** *Priestia koreensis*, levansucrase operon, fructooligosaccharide, enzymatic, sucrose

## Abstract

Levan biopolymer and levan-type fructooligosaccharides (L-FOSs) are β-2,6-linked fructans that have been used as non-digestible dietary fiber and prebiotic oligosaccharides in food and cosmeceutical applications. In this study, we explore the operon responsible for levan and L-FOSs production in *Priestia koreensis* HL12. Presented is the first genomic perspective on sucrose utilization and the levan biosynthesis pathway in this bacterium. Regarding sequence annotation, the putative levansucrase operon responsible for β-2,6-linked fructan was identified in the genome of strain HL12, and comprises *sacB* levansucrase gene belonging to GH68, located adjacent to *levB* endo-levanase gene, which belongs to GH32. Importantly, sugars related with the levan biosynthesis pathway are proposed to be transported via 3 types of transportation systems, including multiple ABC_Sugar_ and glucose/H^+^ transporters, as well as glucose- and fructose-specific PTS systems. Based on product profile analysis, the HL12 strain exhibited high efficiency in levan production from high sucrose concentration (300 g/l), achieving the highest yield of 127 g/l (equivalent to 55% conversion based on sucrose consumption), together with short-chain L-FOSs (DP3-5) and long-chain L-FOSs with respective size larger than DP6 after 48 h incubation. These findings highlight the potential of *P. koreensis* HL12 as a whole-cell biocatalyst for producing levan and L-FOSs, and underscore its novelty in converting sugars into high-value-added products for diverse commercial and industrial applications.

## Introduction

Levan biopolymer and levan-type fructooligosaccharides (L-FOSs) are non-digestible dietary fiber and prebiotic oligosaccharides with various applications in the nutritional, pharmaceutical, and cosmeceutical sectors [1-3]. Levan is a long-chain fructo-conjugated biopolymer composed of β-2,6 linkages in the backbone, with rare β-2,1 branch chains, while L-FOSs represent oligomers with degrees of polymerization (DP) ranging from 2-12. Mostly, levan and L-FOSs are produced by a variety of microorganisms via levansucrase-catalyzed transfructosylation using sucrose as the substrate [3]. Bacterial levan can be extracellularly produced by various bacterial genera, including *Gluconobacter*, *Lactobacillus*, *Bacillus*, *Leuconostoc*, *Zymomonas* , and *Halomonas* [4-13].

Levan-type polymer is synthesized by fructosyl-transferases belonging to the GH-J clan, which includes the two glycoside hydrolase families GH32 and GH68, as shown in the Carbohydrate-Active enZYmes database [14, 15]. Levansucrase (SacB, EC 2.4.1.10), belonging to GH68 and referred to as sucrose:fructan 6-fructosyltransferase (6-SFT), is responsible for both the hydrolysis of sucrose molecules and the catalysis of transfructosylation reactions utilizing either a sucrose molecule or fructooligosaccharides as an acceptor to create β-2,6-linked polymer. It is classified into GH68 together with inulosucrase (EC 2.4.1.9) and β-fructofuranosidase (EC 3.2.1.26). Microbial SacB enzyme is an extracellular levansucrase secreted via the Sec secretion pathway. Meanwhile, endo-levanase (LevB, EC 3.2.1.65), also known as β-2,6-fructanase, catalyzes the hydrolysis of β-2,6 linkage inside the levan backbone, creating oligomers of L-FOSs with different chain lengths. According to amino acid classification, endo-levanase is categorized into glycoside hydrolase family 32 (GH32) along with invertases (EC 3.2.1.26), endo-inulinases (EC 3.2.1.7), fructan β-2,6-fructosidase (EC 3.2.1.154), and various homologous enzymes [16, 17]. *sacB* and *levB* genes, which are important for levan production and degradation, are mostly found in operons, however the number of other related genes and their location in the genome vary among microbial strains. For example, genes involved with levan synthesis and hydrolysis in *Bacillus subtilis* are identified in two operons: levansucrase operon (*sacB*, *levB*, and *yveB* genes) and levanase operon (*sacC*, *levR*, *levD*, *levE*, *levF*, and *levG* genes)[1]. In case of *Cellulosimicrobium cellulans* MP1, levansucrase (*ls* gene) and levan-degrading enzymes (*levB*, *sacC1* and *sacC2* genes) were located in a single operon (*sacC1*-*levB*-*sacC2*-*ls* operon) [18].

Due to the critical roles of sugars and other carbohydrates in bacterial cellular metabolism, these molecules are imported via three major types of sugar transporters depending on their specificity: ATP binding cassette (ABC) transporters, ion gradient major facilitator superfamily (MFS) transporters, and PEP-dependent phosphotransferase system (PTS) transporters [19-21]. In the case of ABC transporters, ATP is used to generate energy for sugar import and export, while the MFS superfamily consists of bacterial sugar transporters, such as glucose (GLUT) transporters. The PTS is a translocation system that transports sugars to their phosphorylation state. Basically, the PTS system consists of phosphotransferase proteins (EI and HPr) and sugar-specific enzyme II complexes (IIA, IIB, and IIC); however, the number of components differs according to bacterial species. For example, glucose transport in *B. subtilis* depends on at least two different systems, including glucose-specific PTS and hexose/H1 symporter [22-24].

In this study, our primary objective was to first identify and characterize a novel levan-producing bacterial strain, *Priestia koreensis* HL12, emphasizing its exceptional ability to produce L-FOSs and levan under high sucrose concentrations. By exploring the genomic perspective and production capabilities of *P. koreensis* HL12, we aim to contribute to the discovery and in-depth understanding of genomic factors influencing the levan production of superior microbial strains suitable for the industrial synthesis of L-FOSs and levan. The significance of our findings lies in the potential of this strain to provide a new avenue for efficient production of these valuable bioproducts, and lead to broader biotechnological application.

## Materials and Methods

### Chemicals

Mono- and di-saccharide standards (glucose, fructose, and sucrose) were purchased from Sigma-Aldrich (USA). Fructooligosaccharides (1-Kestose, 1,1-Kestotetraose, 1,1,1-Kestopentaose, levanbiose, and levantriose) were purchased from Megazyme (Ireland). Yeast extract and peptone were purchased from Difco (USA). All other analytical grade chemicals were purchased from Sigma-Aldrich and Merck (Germany).

### Sucrose Utilization Screening and Species Identification Using 16S rRNA Sequencing Analysis

The bacterial strain *P. koreensis* HL12 (formerly designated as *Bacillus koreensis*) was previously isolated from soil attached to sago palm root (*Cycas revoluta*) [25]. The strain’s sucrose-utilizing capability was evaluated by cultivation in YPS agar plate (5 g/l yeast extract, 5 g/l peptone, and 100 g/l sucrose) at 30°C for 24 h. The bacterial identification was performed by 16S rDNA gene sequence analysis using Prok_BSF_8/20 (5’-AGAGTTTGATCCTGGCTCAG-3’) and Prok_REVB (5’-GGTTACCTTGTTACGACTT-3’) primers. The 16S rDNA gene was amplified using Phusion High-Fidelity DNA Polymerase (Thermo Fisher Scientific, USA) under the following conditions: 95°C for 5 min; 25 cycles of 95°C for 30 s, 55°C for 30 s, and 72°C for 2 min; and a final extension at 72°C for 10 min. The PCR product was purified using the GeneJET Gel Purification Kit (Thermo Fisher Scientific) and cloned into pJET1.2/blunt vector (Thermo Fisher Scientific). The 16S rDNA gene was subsequently sequenced by 1^st^ BASE (Malaysia). The sequence was analyzed by BLAST search against the NCBI database (http://www.ncbi.nlm.nih.gov/BLAST). The evolutionary analysis was conducted using the MEGA11 program [26] using the neighbor-joining method [27] with bootstrap test (5000 replicates) [28]. The evolutionary distances were computed using the Kimura 2-parameter method [29].

### Genome Sequence Analysis and Annotation

Genomic DNA of *P. koreensis* HL12 was extracted using the GeneJET Genomic DNA Purification Kit (Thermo Fisher Scientific,), and the concentration of the extracted genomic DNA was quantified using the NonoDrop 2000 spectrophotometer (Thermo Fisher Scientific). The genome sequencing of *P. koreensis* HL12 was performed through the Illumina 150 PE platform (Novogene, China). Raw paired-end (PE) reads were initially performed for quality control by removing low-quality sequences (average Phred Quality score < 25) and the barcode and adapter sequences were trimmed using FASTP [30]. Next, the cleaned sequences were subjected to *de novo* genome assembly via SPAdes [31] with a minimum contig length of 500 bps. The completeness of bacterial orthologous genes and the contamination of the assembled genome were assessed by BUSCO [32] and CheckM [33], respectively. The assembled contigs were then subjected to structural and functional annotation using the Rapid Annotation using Subsystem Technology (RAST) [34]. All potential carbohydrate-active enzymes were predicted using dbCAN2, a web server for automated carbohydrate-active enzyme (CAZyme) annotation [35], funded by the National Science Foundation (DBI-1933521). Taxonomic classification was conducted by calculating the Tetra Correlation Search (TCS) against the entire genomes reference database (GenomesDB) using JSpeciesWS Online Service [36]. The Gene Ontology (GO) functional annotations of all open reading frame (ORF) sequences were assigned using PANNZER2 to describe the molecular functions, cellular components, and biological processes of genes in the genome [37]. The Clusters of Orthologous Genes (COGs) database was annotated toward the EggNOG v5.0 public database for functional annotation and protein classification from the genome sequence based on the orthology concept [38]. For protein structure annotation, the conserved domain was analyzed by the NCBI Conserved Domains Database (CDD) [39]. The signal peptide involved in protein secretion pathway was analyzed by SignalP 6.0 [40]. The promoter controlling levansucrase operon was predicted using Time-delay neural networks (TDNN) [41].

### Levan-Type Fructooligosaccharides (L-FOSs) and Levan Production

*P. koreensis* HL12 was cultured in LB broth (5 g/l yeast extract, 5 g/l sodium chloride, and 10 g/l peptone) at 30°C with shaking at 200 rpm for 24 h. The 1% (v/v) culture broth was transferred into 50 ml of SUC medium (1.5 g/l ammonium sulfate, 7.2 g/l dipotassium phosphate, 0.2 g/l magnesium sulfate, 200 or 300 g/l sucrose) at 30°C with shaking at 200 rpm for 48 h. The bacterial cell was precipitated by centrifugation at 8,000 ×*g* at room temperature for 5 min. The sugar and L-FOS profile in the supernatant was analyzed using Thin-Layer Chromatography (TLC) and High-Performance Liquid Chromatography (HPLC). The levan polymer was precipitated by absolute ethanol at a 1:3 supernatant:ethanol ratio. The precipitated levan was collected by centrifugation at 12,000 ×*g* at room temperature for 5 min, and subsequently dried using a heating box at 40°C for 24 h.

### Product Profile Analysis Using TLC and HPLC

The sugar and L-FOSs profile was analyzed by Thin-Layer Chromatography (TLC) using TLC Silica Gel 60 F_254_ (Merck, Germany) in a mixture of mobile phase comprising n-butanol, acetic acid, and distilled water at a ratio of 5:2:3. The spot was visualized by developing- solution containing 95% (v/v) absolute ethanol, 5% (v/v) sulfuric acid, and 0.1% (w/v) orcinol. The TLC plate was dried and heated until the spots appeared. For HPLC analysis, the sample was dissolved in 65% (v/v) acetonitrile and subsequently analyzed using a Shodex Asahipak NH2P-50 4E column at 40°C equipped with RID detector using 65% acetonitrile as a mobile phase with a flow rate of 0.7 ml/min.

## Results and Discussion

### Species Identification of Strain HL12 and Its Sucrose Utilization Potential

**Fig. 1:**
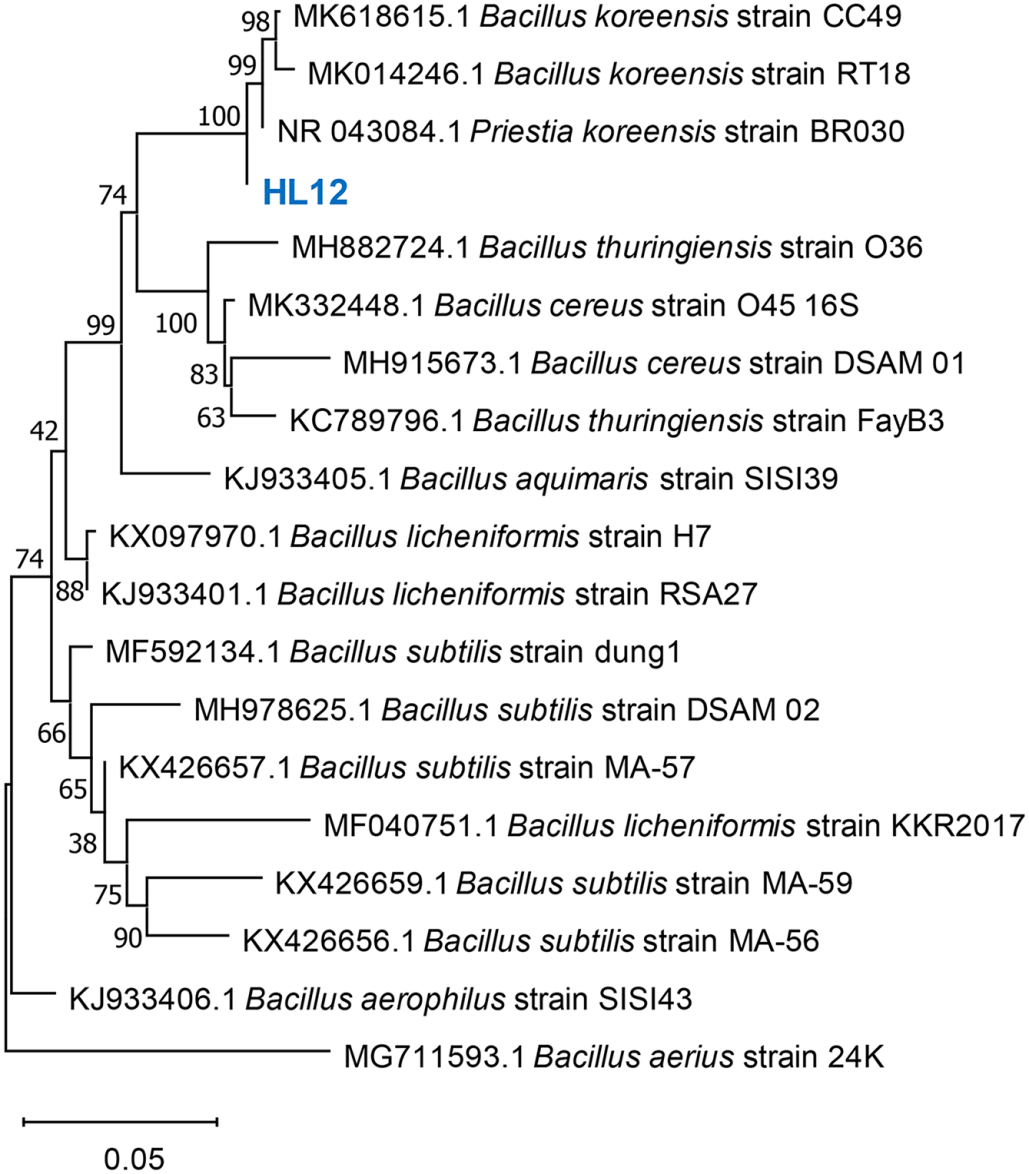
Evolutionary relationships between *P. koreensis* HL12 and other related strains from genus *Bacillus*. The phylogenetic tree was constructed using MEGA11 based on full-length of 16S rDNA using the neighbor-joining method. The evolutionary distance was computed using the Kimura 2-parameter together with bootstrap test (5000 replicates).

Screening of fructan-producing bacteria from soil attached to sago palm root was carried out on YPS agar plate supplemented with 100 g/l sucrose as the sole carbon source. The bacterial strain HL12 (previously designated as *B. koreensis*) exhibited mucous and pale-yellow characteristic representing sucrose-utilizing potential after a 3-day incubation period. Regarding molecular identification, 16S rDNA sequencing indicated that the isolate HL12 is closely related to *Priestia koreensis* strain LQ38 with 99.85% similarity. The evolutionary analysis using a neighbor-joining phylogenetic tree revealed a close relationship between HL12 strain and *Bacillus koreensis* CC49, *Bacillus koreensis* RT18, and *Priestia koreensis* BR030 (Fig. 1). In addition, to the confirm species identification, taxonomic classification based on genome sequence analysis using Tetra Correlation Search (TCS)[36] revealed strain HL12 to be closely related with *Priestia koreensis* DSM 16467, with Z-score 0.99889, followed by *Halalkalibacterium ligniniphilum* L1 (Z-score 0.93632), *Alkalihalobacillus okhensis* Kh10-101 (Z-score 0.93328), and *Bacillus coahuilensis* p1.1.43 (Z-score 0.93033). Based on the strong phylogenetic and conserved signature indels (CSIs) in the protein sequence analysis, *B. koreensis* was reorganized as novel genera with the name *Priestia* gen. nov [42]. In summary, the HL12 strain was reclassified as *P. koreensis* species.

### Mining of Sucrose Utilization-Related Enzymes in *P. koreensis* HL12 Genome

**Fig. 2:**
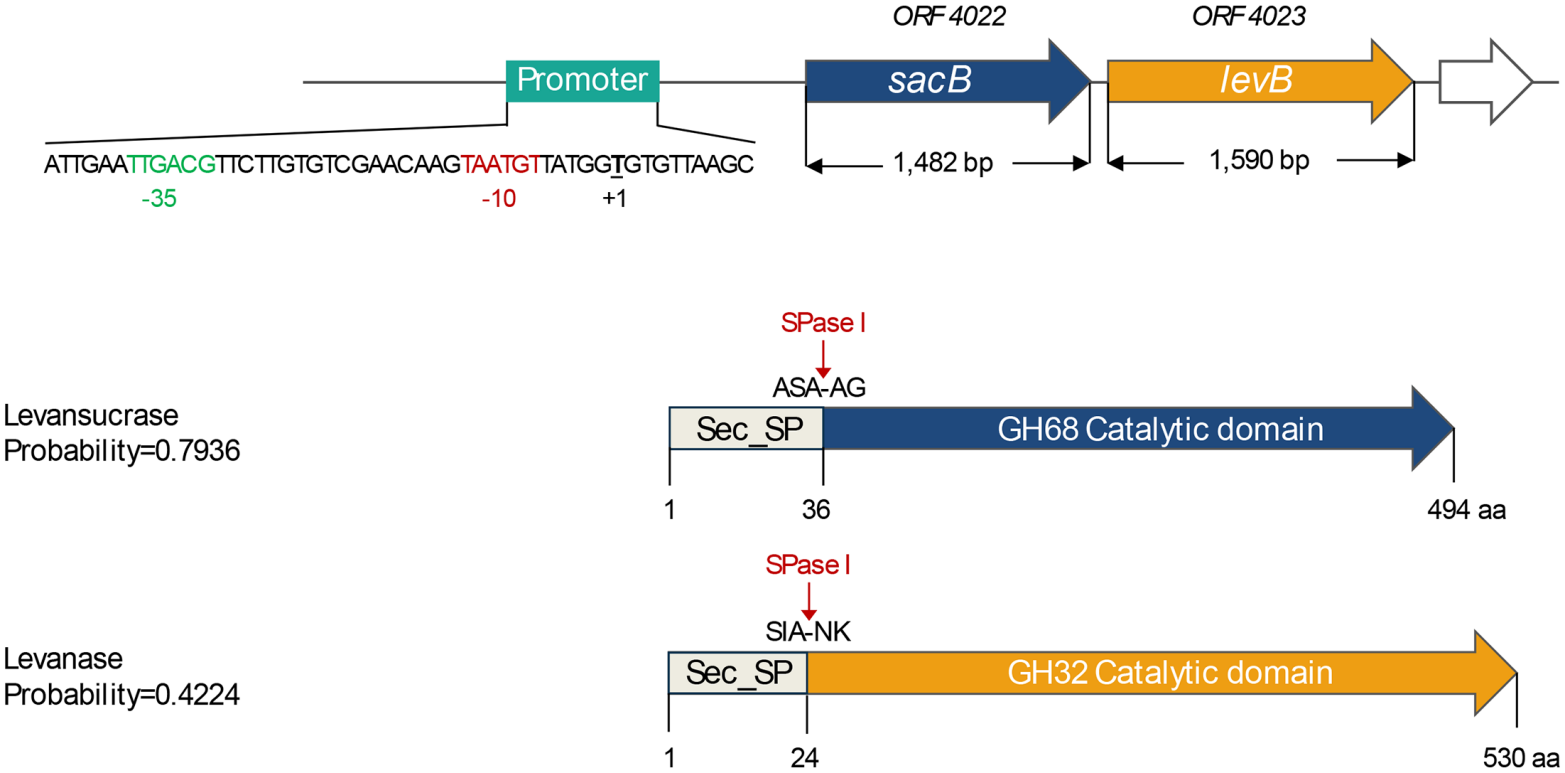
Gene organization of levansucrase operon identified in the *P. koreensis* HL12 genome. The *sacB* and *levB* genes represent levansucrase and endo-levanase, respectively.

Next, the genes encoding the enzymes related to sucrose utilization and fructan biosynthesis in *P. koreensis* HL12 genome were analyzed. Regarding carbohydrate-active enzymes (CAZymes) predicted by dbCAN2, which is a web server for automated carbohydrate-active enzyme annotation, the putative levansucrase operon comprising 1,482 bp of levansucrase gene (*sacB*) (*ORF 4022*) belonging to glycoside hydrolase family 68 (GH68) clustered with 1,590 bp of endo-levanase gene (*levB*) (*ORF 4023*) belonging to glycoside hydrolase family (GH32) was found in the genome of strain HL12 (Fig. 2). The upstream sequence of the operon was predicted as the putative promoter comprising -35 and -10 region with the highest score of 0.96. However, the upstream and downstream ORFs of the putative operon are uncharacterized hypothetical proteins. Moreover, in the absence of inulin-forming enzyme inulosucrase (ISase) in the genome, HL12 is suggested to be a potential whole-cell catalyst for the majority of levan-type fructan production.

Levansucrase (EC 2.4.1.10), also referred to as beta-D-fructofuranosyl transferase, is an enzyme that facilitates the transfer of the sucrose fructosyl group in creating β-2,6-linked oligosaccharides and levan chains. Many studies reported that the enzymes belonging to the GH68 family are present in bacterial genera that include *Acetobacter*, *Bacillus*, *Clostridium*, *Geobacillus*, *Lactobacillus*, *Leuconostoc*, *Pseudomonas*, *Zynomonas*, *Erwinia*, *Gluconobacter*, *Gluconacetobacter*, and *Halomonas* [3, 9, 11, 43-52], while endo-levanase (EC 3.2.1.65) was classified into the GH32 family. Our previous study indicated that recombinant endo-levanase (LevBk) from the HL12 strain hydrolyzes timothy grass levan (β-2,6-linked fructan) to generate levan-type fructooligosaccharides (L-FOSs) with levanbiose (DP2) and levantriose (DP3) predominantly [53]; however, no hydrolytic activity toward inulin (β-2,1-linked fructan) was detected. In addition, no apparent homologs of exo-levanase (*sacC* gene) have been identified in the chromosome of strain *P. koreensis* HL12.

Genes involving levan synthesis and degradation of *B. subtilis* were identified in two operons containing both levansucrase operon with *sacB* and lev gene (endo-levanase) and exo-levanase operon with *sacC* gene [54, 55]. Based on bioinformatic analysis, both *sacB* gene encoding levansucrase and *sacC* gene encoding exo-levanase were found from several levan-producing bacteria, for instance, *Paenibacillus polymyxa* 2020 [56], *C. cellulans* MP1 [57], *Zymomonas mobilis* [58], and *B. subtilis* [59]. Due to high sucrase activity, SacC of *Z. mobilis* plays a major role in sucrose hydrolysis, with >70% of total sucrase activity, which is higher than that of SacB. In addition, SacC exo-levanase can degrade levan into monomeric fructose [54, 59]. Therefore, the construction of a *Z. mobilis* *sacC* mutant can improve the efficiency of levan production [60].

Based on peptide prediction analysis, SacB and LevB from *P. koreensis* are composed of Sec signal peptide with 36 and 24 N-terminal amino acids (Fig. 2) [53]. The SacB *P. koreensis* signal peptide corresponds to several gram-positive levan-producing bacteria, such as *B. subtilis*, *Bacillus amyloliquefaciens*, *Streptococcus salivarius*, and *Lactobacillus panis* [61-64], resulting in the secretion of levansucrase after cleavage. In contrast, in *Gluconobacter*, *Z. mobilis* and *Pseudomonas syringae*, which are gram-negative, levansucrase was secreted using a signal peptide-independent pathway [65-67]. The alteration of levansucrase signal peptide by protein engineering influence on the levansucrase secretion thus led to improving the yield of levan production [68, 69].

### Sugar Transport Systems in *P. koreensis* HL12 Genome

**Fig. 3:**
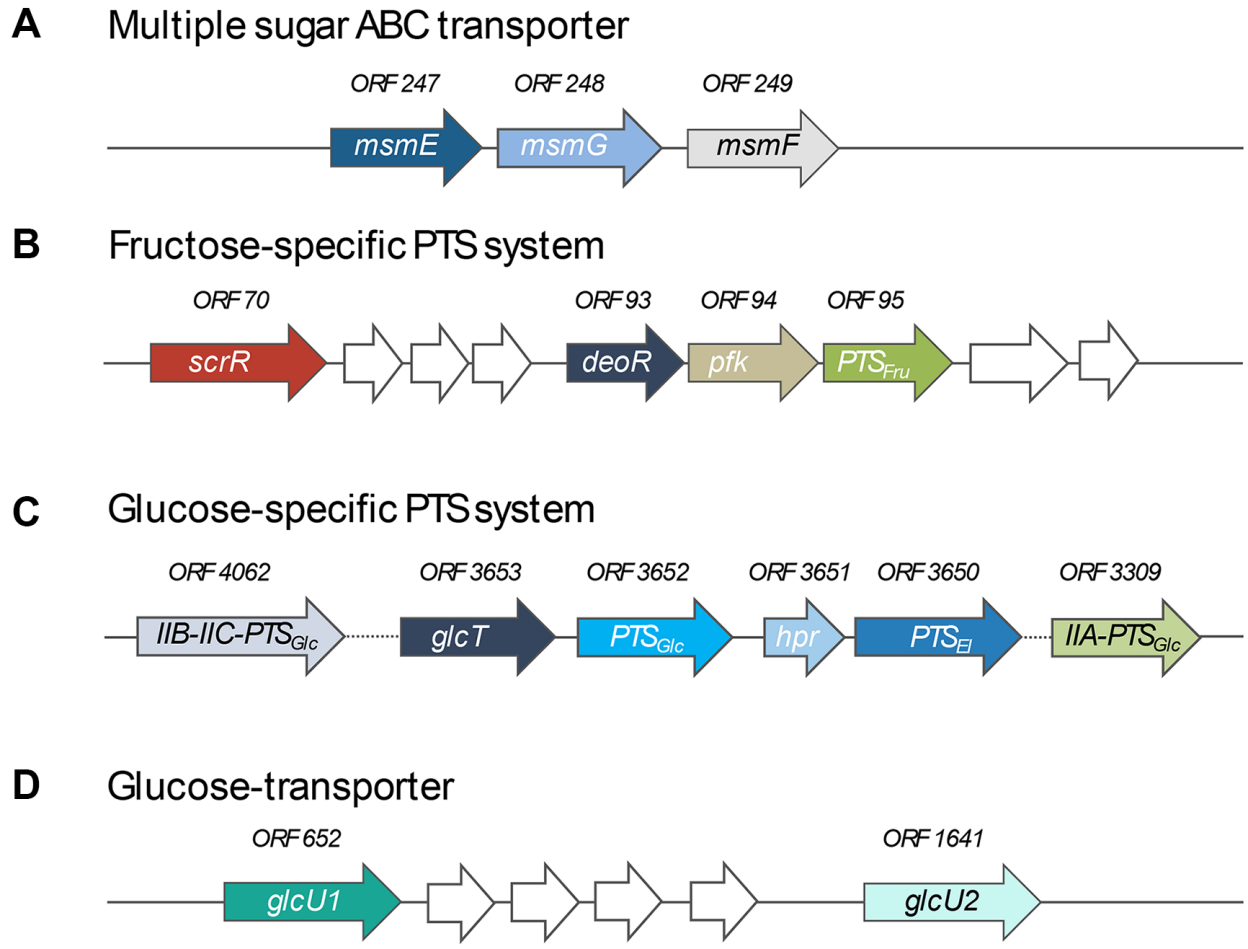
Gene organization within clusters involved in sugar transport systems for sucrose utilization and L-FOSs biosynthesis identified in *P. koreensis* HL12 genome. (**A**) Multiple sugar ABC transporter comprising *msmE*: substrate-binding protein MsmE, *msmG*: permease protein MsmG, and *msmF*: permease protein MsmF. (**B**) Fructose-specific PTS system comprising *scrR*: sucrose operon repressor ScrR, *deoR*: transcriptional repressor of the fructose operon, *pfk*: 1-phosphofructokinase, and *PTS_Fru_*: fructose-specific IIA, IIB, and IIC components. (**C**) Glucose-specific PTS system comprising *glcT* gene: PtsGHI operon antiterminator, *PTS_Glc_*: glucose-specific IIA, IIB, and IIC components, *hpr*: HPr phosphocarrier protein, *PTS_EI_*: EI component, *IIB*-*IIC*-*PTS_Glc_*: maltose and glucose-specific IIC and IIB components, and *IIA*-*PTS_Glc_*: glucosespecific IIA component. (**D**) Glucose-transporter comprising *glcU1* and *glcU2*: glucose/H^+^ symporters.

Regarding *in silico* analysis, gene clusters encoding sugar transporters related with sucrose utilization and levan biosynthesis were identified in the *P. koreensis* HL12 genome (Fig. 3). The HL12 strain has evolved three different sugar transporting systems for sucrose, glucose, and fructose, namely (1) multiple sugar ABC transporter, (2) proton-coupled sugar transporter, and (3) phosphoenolpyruvate-dependent sugar phosphotransferase systems (PTS). Firstly, a gene cluster consisting of *msmE* (*ORF 247*), *msmG* (*ORF 248*), and *msmF* (*ORF 249*), encoding substrate-binding protein MsmE, permease protein MsmG, and permease protein MsmF, respectively, in a multiple sugar ABC transporter system, was identified in the HL12 genome. Many studies reported that the multiple sugar metabolism (Msm) ABC transporter of *Escherichia coli*, *Streptococcus mutans*, and *Thermus thermophilus* HB8 has demonstrated the capability to uptake sucrose, glucose, and fructose from the environment [70-72]. Secondly, two gene clusters encoding fructose-specific and glucose-specific PTS transporting systems were identified. Basically, many sugars are transported by PTS systems in a phosphorylation state via a multicomponent system of phosphotransferases. For fructose-specific PTS systems, the gene cluster comprises *PTS_Fruc_* gene (*ORF 95*) encoding fructose-specific IIA, IIB, and IIC components and *pfk* gene (*ORF 94*) encoding 1-phosphofructokinase (EC 2.7.1.56), which are located downstream of the DeoR-type putative transcriptional regulators (*deoR* gene, *ORF 93*). This indicates that the expression of *PTS_Fruc_* and *pfk* genes are probably regulated by the putative DeoR, which was previously reported as a transcriptional regulator involved in regulating the fructose-PTS cluster genes in *Corynebacterium glutamicum* ATCC 13032 [73]. In addition, *scrR* (*ORF 70*), encoding a sucrose operon repressor (LacI family), was also found in the HL12 genome; however, the function of the ScrR protein remains unidentified due to its distant location from the genes involved in the sucrose utilization pathway. In the glucose-specific PTS gene cluster, we found *glcT* gene (*ORF 3653*) encoding ptsGHI operon antiterminator, *PTS_Glc_* gene (*ORF 3652*) encoding glucose-specific IIA, IIB, and IIC components, *hpr* gene (*ORF 3651*) encoding HPr phosphocarrier protein, and *PTS_EI_* gene (*ORF 3650*) encoding EI component. Importantly, *IIB-IIC*-*PTS_Glc_* (*ORF 4062*) encoding maltose- and glucose-specific IIC and IIB components, and *IIA*-*PTS_Glc_* (*ORF 3309*) encoding glucose-specific IIA component were identified distant from *PTS_Glc_* gene cluster. Lastly, a single open reading frame, *ORF 652* and *ORF 1641*, encoding two glucose/H^+^ symporters (glucose uptake protein, GlcU) involved in glucose internalization, was also found. Basically, the GlcU transporter protein has been reported as a non-PTS pathway significantly involved in the transport of glucose in various bacterial species, including *Streptococcus pyogenes*, *Lactococcus lactis*, and *Staphylococcus xylosus* [74-76].

### Short-Chain L-FOSs and Levan Polymer Production from Sucrose

**Fig. 4:**
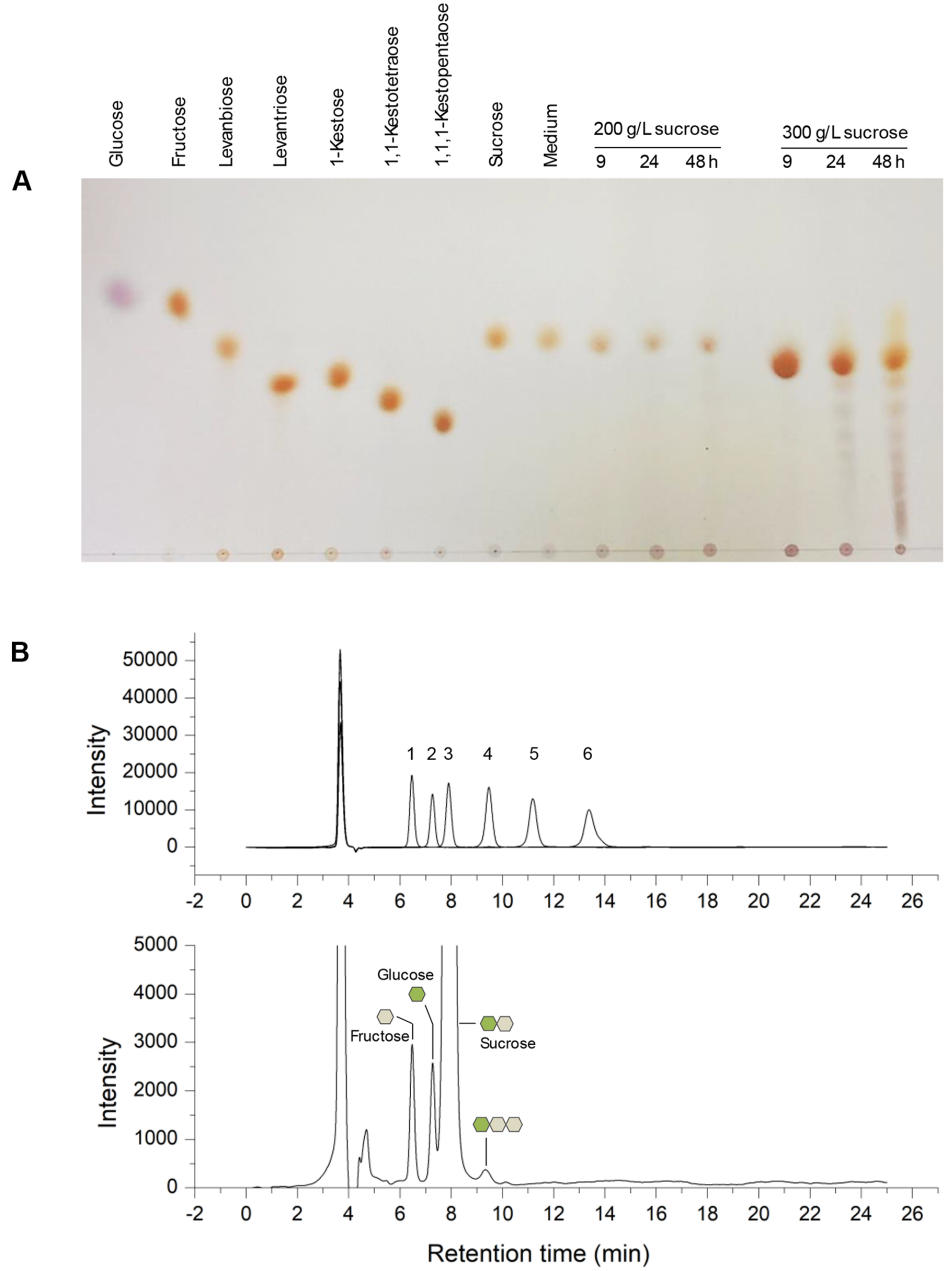
Analysis of sugar profile of L-FOSs produced by *P. koreensis* HL12. The HL12 strain was cultivated in sucrose-rich medium at 200 and 300 g/l sucrose initial concentration at 30°C for 9, 24, and 48 h. (**A**) Sugar profile from TLC analysis (**B**) Chromatogram from HPLC analysis of HL12 cultured in 300 g/l sucrose at 48 h comparing with sugar standards as follows: 1: fructose, 2: glucose, 3: sucrose, 4: 1-Kestose, 5: 1,1-Kestotetraose, and 6: 1,1,1-Kestopentaose.

Regarding genome analysis, enzymes related to levan biosynthesis and sugar transport are present in the *P. koreensis* HL12 genome; however, the capability of the *P. koreensis* species to produce levan-type fructooligosaccharides and levan has not yet been investigated. Therefore, the HL12 strain was cultivated in medium containing different initial sucrose concentrations as a sole carbon source (200-300 g/l). The chain-length and product profile of L-FOSs was preliminarily analyzed using Thin-Layer Chromatography (TLC). According to our findings, L-FOSs and levan production depended on cultivation time and sucrose concentration. The levan content produced by the HL12 strain from 300 g/l was 127 g/l after 48 h incubation. Regarding TLC analysis, the polymer produced from sucrose using *P. koreensis* HL12 was digested into various chain-length oligosaccharides that correspond to hydrolysis of timothy grass levan by recombinant endo-levanase (LevBk) from *P. koreensis* HL12 at 45°C for 24 h [53]. These results demonstrated that the polymer produced from sucrose using the whole-cell biocatalysts *P. koreensis* HL12 is high-molecular-weight levan (Fig. S1). Only a small number of L-FOSs were produced in 200 g/l sucrose after 48 h incubation (Fig. 4A). Meanwhile, at 300 g/l initial sucrose concentration, short-chain L-FOSs (DP3-5) and long-chain L-FOSs with respective size larger than DP6 were produced at 24 and 48 h, and levan polymer in the stationary phase was detected. However, a large quantity of sucrose (68 g/l) remained in the production medium. These findings corresponded with the results from HPLC analysis showing that sucrose was hydrolyzed into fructose and glucose, and a small amount of 1-Kestose (GF_2_) was also generated via transfructosylation reaction. Due to a low concentration of L-FOSs with more than DP3 produced in the reaction, however, they could not be detected using the HILIC mode of HPLC technique (Fig. 4B).

**Fig. 5:**
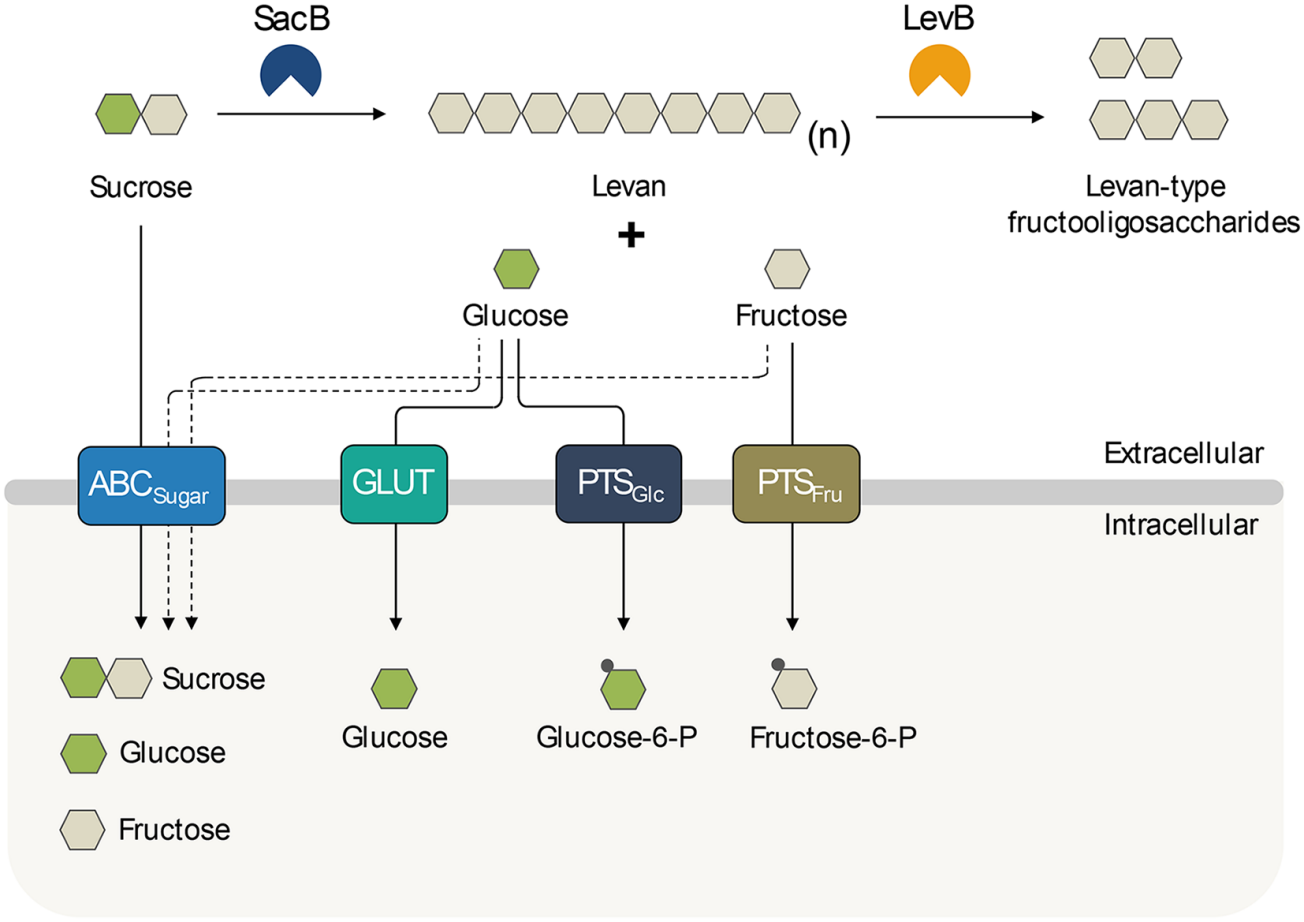
Proposed mechanism for levan-type fructooligosaccharides (L-FOSs) biosynthesis and sugar transport pathways from *P. koreensis* HL12 based on genomic information. ABC_Sugar_: multiple sugar ABC transporter, GLUT: glucose/H^+^ transporter, PTS_Glc_: glucose-specific PTS system, and PTS_Fru_: fructose-specific PTS system.

Our findings in this work propose a β-2,6 fructan biosynthesis pathway (Fig. 5) whereby levan polymer and long-chain L-FOSs are synthesized from sucrose via levansucrase (SacB) reactivity, and subsequently degraded by endo-levanase (LevB) to generate short-chain L-FOSs. Furthermore, the released glucose and fructose from the hydrolytic activity of levansucrase can be transported into the cell through ABC, glcT, PTS_Glc_, and PTS_Fru_, which suggests they are used in a metabolic pathway. An understanding of the interrelation between levan-type biosynthesis and metabolic processes is useful for microbial host transformation via metabolic systems engineering and robust whole-cell factories used for efficient overproduction [77].

However, a lack of the exo-levanase (SacC) operon responsible for long-chain levan hydrolysis leads to the formation of only a small amount of fructose in the HL12 strain. Exploring new strains with balance between exo-and endo-levanase activity is necessary for the development of FOSs and levan production. According to the genome and product profile analysis, HL12 is reported here as having a genome that lacks exo-levanase gene, and thus sucrose conversion is done by cooperation of levansucrase and endo-levanase. Therefore, HL12 can be considered as a novel potential strain for FOS and levan production using high concentration of sucrose, with release of low amounts of by-product fructose and glucose. For levansucrase operon of *B. subtilis*, the action of enzyme encoded by *levB* gene provokes the intrinsic exo-levanase activity of levansucrase encoded by *sacB* gene resulting in the increase of released fructose [54, 78].

Sucrose concentration is related to the growth of microorganisms and the production of levan polymer, depending on the strain and fructosyltransferase activity. Previous reports have identified limitations of microbial whole-cell biocatalyst for levan production, including low conversion yield, even when using high concentrations of sucrose, such as with *Z. mobilis*, *Tanticharoenia sakaeratensis*, *Bacillus aryabhattai*, *B. amyloliquefaciens* and *B. subtilis*, which produced conversion yields ranging from 4.8-13.4% from sucrose concentrations of 162 and 299 g/l [54, 79]. However, various other microbes are capable of synthesizing low amounts of levan (8.8-15.4 g/l) in growth media containing low concentrations of sucrose (50-60 g/l) with long incubation times ranging from 60 to 160 h. For instance, *Acetobacter xylinum*, *Halomonas smyrnensis*, and *Pseudomonas fluorescens* have demonstrated this capability [79]. This variation could be influenced by the different abilities of microbial homeostasis in responding to osmotic stress, which is crucial for cell survival. Furthermore, some attractive microbial strains have shown high potential for levan and L-FOSs production, achieving high yields from high sucrose concentrations. Despite the characterization of various levan producers, few microbes with high levan yields have been identified, including *B. subtilis* natto CCT7712 [80], *Bacillus methylotrophicus* SK 21.002 [81], *Z. mobilis* CCT 4494 [82], which achieved conversion yields of 27.9, 33.3 and 37.5% respectively, from sucrose concentrations of 400 and 300 g/l, compared to *P. koreensis* HL12, which achieved a 42.6% conversion yield from 300 g/l of sucrose. Therefore, based on its genomic profile, media composition and conversion yield, *P. koreensis* HL12 could be considered a novel and effective levan producer with economic viability for the biotechnology industry.

## Conclusion

This study emphasized the potential of *P. koreensis* HL12 as a novel L-FOSs and levan-type fructan-producing bacteria due to the presence of levansucrase-endo-levanase operon without exo-levanase operon in its genome. Moreover, *P. koreensis* HL12 has attracted interest due to its ability to produce high amounts of levan with low glucose-fructose by-product using high concentration of sucrose. Based on genomics organization analysis, the association of sucrose-utilizing related enzymes and sugar transport systems involved in levan production of *P. koreensis* were first reported in this study. The findings here provide valuable insight into the molecular mechanisms underlying sucrose utilization, levan synthesis and degradation, which are useful for development of levan production using *P. koreensis* HL12 as a whole-cell biocatalyst.

## Supplemental Materials

Supplementary data for this paper are available on-line only at http://jmb.or.kr.
