# Supplementary material for: Exploring Levansucrase Operon Regulating Levan-Type Fructooligosaccharides (L-FOSs) Production in Priestia koreensis HL12
Source: J Microbiol Biotechnol. 2024 Aug 23;34(10):1959–68. doi: 10.4014/jmb.2404.04043 (PMC11540611; doi:10.4014/jmb.2404.04043)
Supplement: Supplementary file 1 [file jmb-34-10-1959-supple.pdf]

J. Microbiol. Biotechnol. 2024. 34(10): 1959-1968

https://doi.org/10.4014/jmb.2404.04043

Supplementary Figure

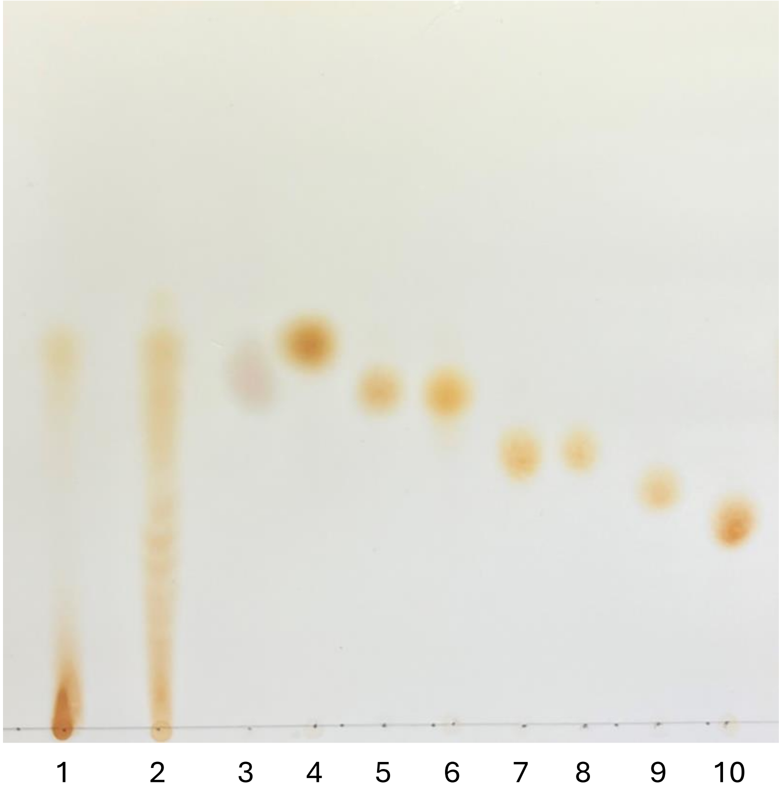

Fig. S1. Analysis of sugar profile obtained from high molecular weight levan hydrolysis. The high molecular weight levan produced by *P. koreensis* HL12 was hydrolyzed by recombinant endo-levanase (LevBk) from *P. koreensis* HL12 at 45°C for 24 h comparing with sugar standards as following, 1: levan produced using *P. koreensis* HL12, 2: levan produced using *P. koreensis* HL12 treated with endo-levanase, 3: glucose, 4: fructose, 5: sucrose, 6: levanbiose, 7: levantriose, 8: 1-Kestose, 9: 1,1-Kestotetraose, and 10: 1,1,1-Kestopentaose
